# RNA-based NGS检测*EML4-ALK*融合V1亚型肺腺癌1例

**DOI:** 10.3779/j.issn.1009-3419.2024.102.23

**Published:** 2024-06-20

**Authors:** Yue XU, Ning MU, Mei LIU, Shengnan WU, Chunhua MA

**Affiliations:** 300121 天津，天津市人民医院肿瘤诊疗中心; Department of Cancer Diagnosis and Treatment Center, Tianjin People's Hospital, Tianjin 300121, China

**Keywords:** 肺肿瘤, 间变性淋巴瘤激酶, 基因融合, 下一代测序, 免疫组化, 靶向治疗, Lung neoplasms, Anaplastic lymphoma kinase, Gene fusion, Next-generation sequencing, Immunohistochemistry, Targeted therapy

## Abstract

肺癌是全球发病率和死亡率最高的恶性肿瘤。对于肺腺癌而言，识别特定的基因突变并给予相应靶向药物可大大提高患者的生存时间。其中，间变性淋巴瘤激酶（anaplastic lymphoma kinase, ALK）融合发生在3%-7%的非小细胞肺癌（non-small cell lung cancer, NSCLC）中。在临床中，多种检测方法可应用于判断ALK融合状态，但存在检测结果假阴性可能。本文回顾性分析1例肺腺癌患者的诊治经过，经多种检测方式判断ALK融合状态，其中，免疫组化（immunohistochemistry, IHC）（Ventana D5F3）、基于RNA的下一代测序（RNA-based next-generation sequencing, RNA-based NGS）检测证实棘皮类微管关联蛋白样4（echinoderm microtubule associated protein like 4, EML4）-ALK融合阳性，而基于DNA的NGS（DNA-based NGS）检测为阴性。本文比较分析判断ALK融合的检测方法，以明确在不同临床案例中选择何种检测方式最准确、最简便，以便于指导后续治疗。

## Abstract (en)

Lung cancer is the malignant tumor with the highest incidence and mortality rate worldwide. For lung adenocarcinoma, identifying specific gene mutations, fusions, and giving corresponding targeted drugs can greatly improve the survival time of the patients. Among them, anaplastic lymphoma kinase (ALK) fusion occurs in 3%-7% of non-small cell lung cancer (NSCLC). In clinical practice, a variety of detection methods can be used to determine the ALK fusion status, but false negative test results are possible. This paper retrospectively analyzed the diagnosis and treatment of a patient with lung adenocarcinoma, judged the ALK fusion status by various detection methods. Among them, immunohistochemistry (IHC)(Ventana D5F3), RNA based next-generation sequencing (RNA-based NGS) confirmed positive echinoderm microtubule associated protein like 4 (EML4)-ALK fusion, while DNA-based NGS was negative. This paper analyzed the detection methods of ALK fusion, in order to clarify which detection method is the most accurate and simple to choose in different clinical cases and guide the subsequent treatment.

间变性淋巴瘤激酶（anaplastic lymphoma kinase, ALK）是肺腺癌重要的驱动基因^[1,2]^，2007年，在肺癌中发现了棘皮类微管关联蛋白样4（echinoderm microtubule associated protein like 4, EML4）-ALK基因融合^[3]^。EML4氨基端的一半与染色体2p内ALK的胞内激酶结构域的融合，导致了嵌合酪氨酸激酶的表达^[4]^。EML4-ALK融合基因在体内外均具有强大的细胞增殖、凋亡和转移等关键生物活性^[5]^，可被ALK激酶抑制剂有效阻断^[6]^。因此在肺腺癌中明确是否存在EML4-ALK融合可为后续靶向治疗提供方向^[7]^。本文报道了1例基于DNA的下一代测序（DNA-based next-generation sequencing, DNA-based NGS）基因检测未发现驱动基因突变的晚期肺腺癌患者，经基于RNA的NGS（RNA-based NGS）基因检测为EML4-ALK基因融合阳性，应用ALK抑制剂后效果良好，为临床选择ALK基因融合的检测方式提供了一定的参考。

## 1 病例资料

患者，58岁，女性，主因“咳嗽3周”于2023年8月24日首次就诊天津市人民医院肿瘤诊疗中心。患者于2023年8月因出现无诱因咳嗽，经当地医院检查考虑为肺部肿瘤，为进一步检查就诊外院肿瘤科，行正电子发射计算机断层扫描（positron emission tomography/computed tomography, PET/CT）检查示：（1）右肺肿物伴代谢增高，考虑肺癌，远端肺组织阻塞性改变；（2）纵隔、右侧内乳区及右肺门淋巴结伴代谢增高，考虑转移可能性大；（3）右侧第6肋骨成骨性骨质破坏伴软组织肿物形成，伴代谢增高，考虑转移。2023年8月16日患者行肺部肿物穿刺活检术，病理提示肺腺癌，免疫组化（immunohistochemistry, IHC）：c-ros原癌基因1酪氨酸激酶（c-ros oncogene 1 receptor kinase, ROS1）（-）、甲状腺转录因子1（thyroid transcription factor-1, TTF-1）（3+）、新天冬氨酸蛋白酶A（new aspartic proteinase A, Napsin-A）（3+）、细胞增殖指数Ki-67（+10%）、P63（3+）、ALK（2+）、CD56（-）、细胞角蛋白7（cytokeratin 7, CK7）（3+）、表皮生长因子受体（epidermal growth factor receptor, EGFR）-L858R（-）、间质上皮细胞转化因子（mesenchymal-epithelial transition factor, MET）（1+）、CK20（-）。病理会诊回报（中国医学科学院肿瘤医院，2023年8月24日）：ALK（未注明克隆号）（阳性）（石蜡切片）。患者既往无吸烟饮酒史，无慢性病及恶性肿瘤家族遗传病史。入院诊断：右肺恶性肿瘤、淋巴结转移、骨转移，肿瘤原发灶-淋巴结-转移（tumor-node-metastasis, TNM）分期：T2N2M1，IVA期。入院后于2023年8月24日行胸部CT（图1）：（1）右肺下叶肿物，符合肺癌表现；（2）双肺多发微结节，随诊复查；（3）纵隔、右肺门及右内乳区多发稍大淋巴结；（4）右侧第6肋骨骨质破坏。因再次预约切病理组织片行ALK检测需要时间，且患者喘息伴骨痛较前加重，于2023年8月25日予静脉化疗1个周期（培美曲塞二钠700 mg d1+卡铂400 mg d1）。2023年9月4日（病理组织石蜡切片）DNA-based NGS基因检测：未检出肿瘤驱动基因突变。因患者为亚裔女性且无吸烟史，根据指南推荐，行免疫组化（immunohistochemistry, IHC）（Ventana D5F3）检测以及（病理组织石蜡切片）RNA-based NGS基因检测。2023年9月11日行IHC（Ventana D5F3）：ALK（+）。2023年9月14日患者第2次入院治疗，完善胸部CT（见图2）：（1）右肺下叶肿物，符合肺癌表现，较前（2023年8月14日）变化不明显；（2）双肺多发结节，随诊复查。余表现同前。病灶无缩小，疗效评估为疾病稳定（stable disease, SD）。结合IHC （Ventana D5F3）法提示ALK（+），予洛拉替尼100 mg qd分子靶向治疗。2023年9月23日（病理组织石蜡切片）RNA-based NGS基因检测提示：EML4-ALK基因融合阳性，V1亚型。2023年10月12日患者第3次入院治疗，行胸部CT评估病灶（图3）：（1）右肺下叶肿物，符合肺癌表现，较前（2023年9月14日）减小；（2）原双肺多发微结节已基本消失；（3）纵隔多发稍大淋巴结，较前减小；病灶缩小明显，疗效评估为部分缓解（partial response, PR），且患者咳嗽症状较前明显缓解，体力状况（performance status, PS）评分为1分，继续洛拉替尼分子靶向治疗。随访至2024年2月，患者一般状况良好，继续口服洛拉替尼分子靶向治疗。

**图1:**
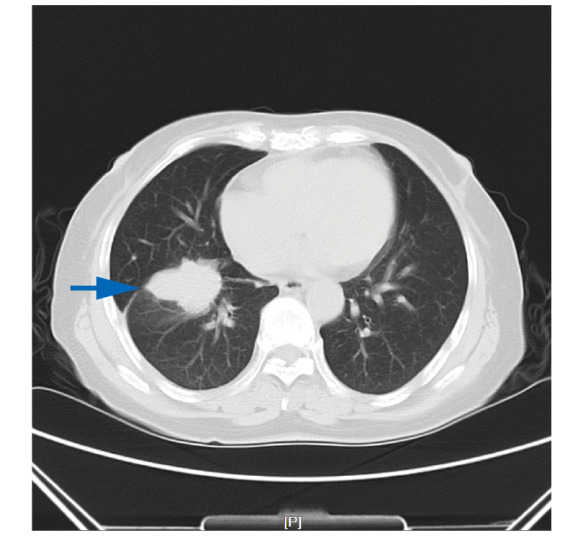
2023年8月24日，患者治疗前右肺下叶占位性病变，大小约4.8 cm×3.2 cm。

**图2:**
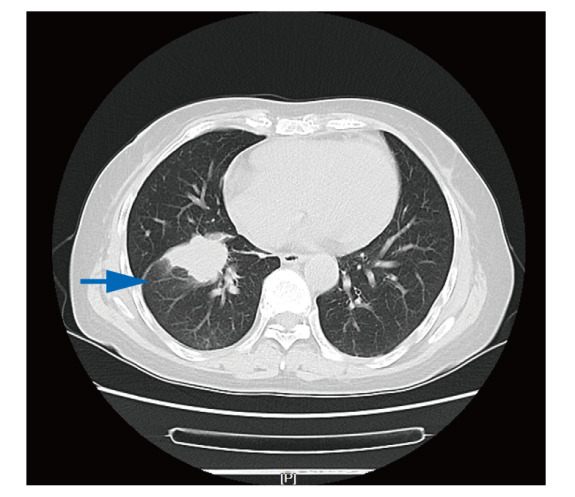
2023年9月14日，静脉化疗1个周期后病变无缩小，大小约4.7 cm×3.5 cm，疗效评估为疾病稳定。

**图3:**
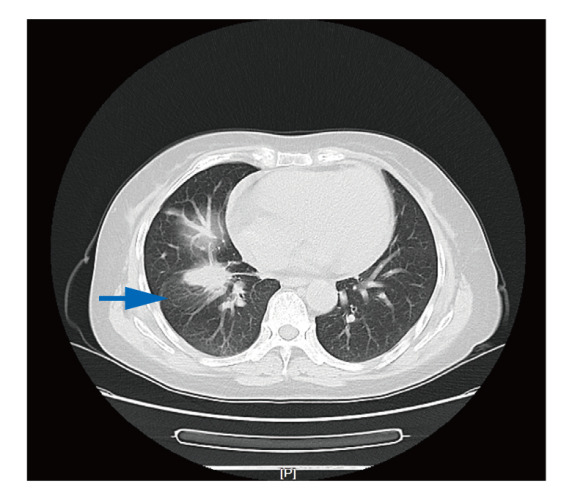
2023年10月12日，洛拉替尼靶向治疗1个周期后病灶明显缩小，大小约3.4 cm×2.5 cm，疗效评估为部分缓解。

## 2 讨论

随着荧光原位杂交（fluorescence in situ hybridization, FISH）和NGS等技术的出现，肿瘤进入精准诊疗时代，肿瘤基因的筛选已成为一种常规的诊断和指导治疗方法^[8,9]^。EML4-ALK融合基因是非小细胞肺癌（non-small cell lung cancer, NSCLC）最重要的致病基因之一^[10,11]^，该融合基因最早由Soda等^[3]^报道，在1例肺腺癌患者的肿瘤组织中观察到一个3926 bp的DNA片段，该片段编码一个由1059个氨基酸组成的蛋白，即融合蛋白EML4-ALK。在Soda等^[5]^的后续实验中发现，将EML4-ALK基因植入正常肺细胞可诱导癌变，提示EML4-ALK具有致癌作用。

目前，常用于检测ALK基因融合的方法包括IHC（Ventana D5F3）、FISH、荧光定量聚合酶链式反应（polymerase chain reaction, PCR）和NGS^[12]^。根据遗传中心法则，基因融合在DNA、RNA水平均可能发生，IHC检测应用抗原抗体反应检测肿瘤中是否存在ALK融合蛋白表达，能够结合形态学与功能学信息，该方法具有简便、经济且高敏感度等优点，是临床中应用最多的检测方法。其中，非IHC（Ventana D5F3）法检测ALK基因融合特异性较低，指南未做推荐。2020年美国国立综合癌症网络（National Comprehensive Cancer Network, NCCN）指南更新推荐：IHC（Ventana D5F3）伴随诊断作为独立ALK检测，无需FISH复核，可直接指导用药^[13]^。与此同时，免疫组化受限于抗体类型限制，且检测类型单一，并不能适用于所有的融合基因突变。FISH检测是既往检测融合变异的“金标准”，利用荧光探针识别异常拷贝数扩增和染色体结构改变，也具有一定的局限性。FISH检测结果的判读存在一定的主观性，依赖于诊断医师的经验与判读水平；无法分辨具体融合类型；具有复杂染色模式的融合或染色体内微缺失形成的融合，可能出现假阴性结果^[14]^。PCR检测可以通过设计针对不同融合变体的引物来区分不同类型的ALK融合基因，可明确融合伴侣，敏感性高，操作简便，但不能完全检测所有融合类型以及新的未知的融合型^[15]^。同时，PCR检测对新鲜肿瘤标本提取的RNA的质量要求较高，但大多数肿瘤标本均在中性甲醛中固定，导致RNA降解，敏感性降低^[16,17]^。随着高通量技术的发展，NGS已越来越多地应用于融合基因的检测，其中，DNA-based NGS是NSCLC临床诊断中优先应用的检测手段，可同时检测突变、融合和扩增，检测范围广，可识别常规检测方法无法检测出的基因融合、突变类型^[18]^。DNA-based NGS也存在局限性，由于内含子区断裂位点不固定，目标基因外显子和内含子区域需设计有效的探针覆盖，否则可能发生漏检，而融合区域的重复序列对探针设计要求高，对检测结果有影响^[19]^。在RNA-based NGS中，成熟的RNA为外显子拼接，断裂位点虽发生于内含子，但拼接位置通常相对固定，干扰较小。对于DNA-based NGS未检出融合基因的情况下，RNA-based NGS能够提高融合基因的检出率^[20]^。NSCLC指南推荐：对于通过广泛的基因检测未发现驱动基因突变的患者（尤其是从不吸烟者），推荐考虑行RNA-based NGS检测，从而最大程度地发现基因融合事件^[21]^。2023年《基于RNA-based NGS检测非小细胞肺癌融合基因临床实践中国专家共识》中指出：相比DNA-based NGS，RNA-based NGS不受内含子影响，可提升融合基因的检出率。建议有条件的医疗机构对NSCLC样本进行一次性同步RNA-based NGS与DNA-based NGS的驱动基因变异（融合/突变）检测^[22]^。

在本病例中，患者经病理组织DNA-based NGS检测未发现驱动基因突变，行IHC（Ventana D5F3）提示ALK（+），病理组织RNA-bases NGS检测提示EML4-ALK基因融合阳性，V1亚型。患者接受洛拉替尼分子靶向治疗，取得较好的临床效果。提示本例融合基因可能发生在RNA层面，或基因在DNA层面融合丰度低，或是存在长内含子或重复序列的重合；这时RNA-based NGS可以有效避免这些融合基因的漏检^[23]^。在斯隆凯特琳癌症中心的一项研究^[24]^中，232例经DNA-based NGS检测缺乏致癌驱动因素改变的肺腺癌，经过RNA测序证实，14%（n=36）的病例存在基因融合或重排阳性。此外，80%的基因融合且接受匹配靶向治疗的肿瘤患者获得了临床获益。需要注意的是，少数肿瘤样本可能存在DNA异常碎裂后重组，导致DNA测序阴性结果而在RNA层面能够检测到的现象。本例单次检测DNA-based NGS结果阴性，也不能排除是假阴性，重测也可能提高阳性率，因此在条件允许的情况下可多次检测，提高准确率。

综上所述，对于ALK基因融合的检测，临床中首先推荐IHC（Ventana D5F3）法检测，因其经济、准确、快捷，但该方法不能区分ALK基因融合亚型。病理组织DNA-based NGS可识别常规检测方法无法检测出的基因融合、突变类型。对于DNA-based NGS检测未检测出驱动基因突变的患者，可以尝试RNA-based NGS检测以最大限度地发现基因融合，最终达到精准识别并指导治疗的目的。
